# Effect of a digital training package on clinical outcomes in Malawi’s index case testing programme: a cluster randomised controlled trial

**DOI:** 10.1136/bmjgh-2025-022563

**Published:** 2026-03-13

**Authors:** Nora Rosenberg, Katie Mollan, Jiayu Wang, Tapiwa Tembo, Mike Chitani, Elizabeth Wetzel, Sarah E Rutstein, Victor Mwapasa, Duncan Phiri, Angella Mkandawire, Vivian Go, Maria Kim, Saeed Ahmed, Katherine R Simon

**Affiliations:** 1 Gillings School of Global Public Health, University of North Carolina at Chapel Hill, Chapel Hill, North Carolina, USA; 2 Center for AIDS Research, University of North Carolina at Chapel Hill, Chapel Hill, North Carolina, USA; 3 School of Medicine, University of North Carolina at, Chapel Hill, North Carolina, USA; 4 Baylor College of Medicine Children’s Foundation Malawi, Lilongwe, Malawi; 5 School of Global and Public Health, Kamuzu University of Health Sciences, Blantyre, Malawi; 6 Baylor College of Medicine Department of Pediatrics, Houston, Texas, USA

**Keywords:** HIV, Africa, Global Health, Cluster randomized trial, Telemedicine

## Abstract

**Introduction:**

Training health workers is a common implementation strategy to expand evidence-based interventions. Digital training is a promising way of reaching more health workers, minimising clinical interruptions and lowering costs, but clinical outcomes are rarely evaluated in low-income settings. Clinical outcomes were evaluated in Malawi’s HIV index case testing programme through the Package of Resources for Assisted Contact Tracing: Implementation, Costs, and Effectiveness study.

**Methods:**

An unblinded cluster randomised controlled trial was conducted in 33 health facilities in two Malawian districts (2022–2023). Facilities were stratified by district and size and randomly assigned 2:1 to receive standard training or standard training plus a digital-based package. The package included asynchronous role-modelling, small-group practice, one-on-one feedback and tablet-guided quality improvement. Participants were lay health workers involved in index case testing. Index clients were people diagnosed with HIV. Contact clients were their partners, children and household members. Five coprimary outcomes, abstracted from programme registers over 1 year, were assessed at the cluster level: index client participation, contacts elicited, contacts tested, new HIV diagnoses and self-test kit provision. Impacts were estimated using negative binomial mixed-effects models (α=0.05).

**Results:**

Clusters were randomly assigned to enhanced (n=11) or standard (n=22) arms and analysed in four 3-month increments (calendar-quarters) over a 1-year period. In each calendar quarter, clusters had a median of 281 potential index clients (IQR 220–427). Significant effects were observed for three primary outcomes: contact client elicitation (RR=1.37, 95% CI 1.10 to 1.71, p=0.006), contact client testing (RR=1.45, CI 1.10 to 1.92, p=0.01) and self-test kit provision (RR=2.29, 95% CI 1.19 to 4.40, p=0.01). Positive, but non-significant effects were observed for index client participation (RR=1.22, CI 0.93 to 1.60, p=0.1) and new HIV diagnoses (RR: 1.28, CI 0.94 to 1.76, p=0.1). No study-related adverse events occurred.

**Conclusions:**

Enhanced digital training positively impacted meaningful clinical outcomes and could be replicated for expansion to other evidence-based interventions.

**Trial registration number:**

NCT05343390.

**WHAT IS ALREADY KNOWN ON THIS TOPIC**

- Health worker training is a cornerstone of health system strengthening in low and middle-income countries (LMICs), with billions invested annually.
- Digital training has been highlighted as promising, but until now, there was no evidence that it improved patient-level or client-level outcomes.

**WHAT THIS STUDY ADDS**

- This is the first study to show that a digital training package directly improved clinical outcomes in a resource-limited setting.
- In Malawi’s HIV index testing programme, digital training increased contacts elicited, HIV self-test kits distributed and contacts tested.
- The package was feasible to implement despite low digital literacy and limited connectivity.

**HOW THIS STUDY MIGHT AFFECT RESEARCH, PRACTICE OR POLICY**

- Demonstrates that well-designed digital training can be practical and effective for frontline health workers in constrained environments.
- Offers a scalable model that can inform future HIV programmes and other health areas in LMICs.
- This research fills a major gap in the global health literature by providing crucial evidence on the impact of digital health worker training on clinical results.

## Introduction

 Health worker training is a common implementation strategy in low and middle-income countries (LMIC).^1^ In recent years, it has accounted for $1.7 (USD) billion annually in development assistance for health, a large share of human resource-related expenditures.^2^ Health worker training is designed to improve health worker knowledge, enhance skills, change health worker practices and ultimately improve clinical outcomes.^3^ However, the relationship between training modalities and downstream clinical outcomes has not been well studied.^4^

Synchronous, centralised, in-person, facilitator-delivered training remains the predominant approach to health worker training in the African region.^5^,^7^ However, this approach has important challenges, including high costs of transport, lodging and daily allowances; disruptions in care when multiple health workers receive synchronous training; variability in fidelity due to inconsistent administration^8^,^10^; difficulty repeating or reviewing content; and difficulty monitoring progress.^11^ Decentralised digitally guided trainings have the potential to address these challenges.^12^,^15^ They can be offered locally on electronic devices, minimising travel-related expenses. They can be scheduled asynchronously, reducing clinical disruptions. Furthermore, content is standardised, reviewable and easily monitored. The WHO has recognised digital approaches, especially those that combine digital and face-to-face learning, as promising.^16^ However, such approaches have rarely been rigorously evaluated for clinical outcomes in LMIC,^17^,^19^ especially among lay health workers.^8^

To address these gaps, we developed and pilot-tested a digital training for improving health workers’ capacity to deliver HIV index case testing services in Malawi.^20^ In a pilot assessment, we observed preliminary improvements in fidelity to index case testing procedures and clinical outcomes.^20^ Given promising preliminary results, we designed the Package of Resources for Assisted Contact Tracing: Implementation, Costs and Effectiveness (PRACTICE study). PRACTICE was a hybrid implementation-effectiveness cluster randomised controlled trial designed to examine implementation, clinical and cost-effectiveness outcomes across two districts in Malawi.^21^ This primary PRACTICE study analysis examines adoption of the implementation package at the health worker level and the impact of the package on five coprimary clinical outcomes: rates of participation by index clients living with HIV, elicitation of contact clients, contact client testing, HIV diagnosis among contact clients and provision of HIV self-test kits to index clients for secondary distribution to contact clients. These outcomes were analysed at the cluster level. Study-related adverse events experienced by health workers were monitored.

## Methods

### Setting

The study was conducted in health facilities in Machinga and Balaka, Malawi that were implementing Malawi’s national HIV programme. These facilities were supported by the Tingathe Program, a US President’s Emergency Plan for AIDS Relief (PEPFAR) implementing partner affiliated with Baylor College of Medicine Children’s Foundation Malawi.^5 22^ In light of Malawi’s formidable human resource shortage in the health sector (<2 clinicians per 100 000 people), a focus of Tingathe is hiring, training and deploying hundreds of lay health workers across multiple districts.^5 22^ These lay cadres conduct a majority of HIV index case testing services nationally.^23^

The study was designed to examine a digital training package for lay health workers. The primary health worker recruitment and enrolment period was May–July, 2022. Enhanced training occurred from August to September 2022. Study follow-up occurred from October 2022 to September 2023.

### The index case testing programme

Aligning with WHO and Malawi guidelines, index case testing involves asking people living with HIV (index clients) to invite their sexual contacts, household members and biological children (contacts) for HIV testing. Index clients select whether they prefer to invite their contacts themselves using a family referral slip (passive referral) or with clinical outreach (assisted referral). These services are offered to two index client populations—people newly diagnosed with HIV in the HIV testing services (HTS) setting and those previously diagnosed with HIV and on antiretroviral therapy (ART) in the HIV treatment setting. At the time of the study, there were many more people in the ART setting, given that most people living with HIV already knew their HIV status; new HIV diagnoses were infrequent.^24^

### Study design and population

We conducted a hybrid implementation-effectiveness cluster randomised controlled trial across health facilities supported by Tingathe in Machinga and Balaka.^21^ The overall study had three sets of aims: (1) implementation, (2) clinical effectiveness and (3) cost-effectiveness. This paper focuses on the study’s five coprimary clinical effectiveness outcomes—indicators along the HIV index case testing cascade. We also report on one implementation outcome (implementation strategy adoption) and adverse events. Other outcomes will be reported separately. The primary study period that we report on in this analysis was 1-year long.

All 34 facilities offering HIV index case testing and supported by Tingathe were eligible and included. Most facilities (n=32) were treated as a single cluster; two similar neighbouring facilities were combined a priori to form a single cluster, providing 33 clusters overall. The 33 clusters were stratified into six randomisation strata based on district (Machinga or Balaka), facility level (hospital, health centre or dispensary) and index case testing performance in the prior year. Within each stratum, the clusters were randomly assigned in a 2:1 ratio (standard:enhanced) by an independent statistician using an algorithm coded in SAS V.9.4 (Cary, North Carolina).

PRACTICE study research staff recruited health workers from May to July 2022. Inclusion criteria for health workers included age ≥18 years, working full-time at one of the participating health facilities and being involved in the HIV index case testing programme. Those who were eligible and interested in participating provided written informed consent. We sought to enrol at least two health workers per cluster with no upper limit.

In accordance with Malawian regulatory guidance, all participants received $10 per study activity. Over the primary study period, health workers received $10 up to 10 times: three times for completing surveys, three times for training activities (enhanced arm) and four times for quarterly data abstraction.

To ensure that the study arm did not influence enrolment decisions, randomisation assignments were revealed to clusters and study staff after health worker enrolment and immediately prior to enhanced training activities. Participants, staff and investigators were not blinded to study arm due to the nature of the implementation strategies. To avoid contamination, the digital training package was only available on study tablets and only used at the facilities. Data analysts were masked to randomisation arm when cleaning and preparing the analytic dataset.

### Standard implementation strategy

In October 2021, the Tingathe programme was at the beginning of a 5-year PEPFAR grant cycle. Many lay health workers from the prior PEPFAR cycle continued and new staff were hired. To orient all lay health workers to the 5-year programme, a 4-day training was offered, including 2 hours devoted to HIV index case testing. This brief training was held offsite, conducted synchronously and offered face-to-face. The training occurred once, was facilitator-delivered and primarily didactic. It provided an overview of index case testing concepts, procedures and counselling approaches. Additionally, the Tingathe programme offered routine supervision and observations and quality improvement activities at all facilities, but these were often not focused on index case testing. These activities were conducted in all 33 clusters.

### Enhanced implementation strategy

**Figure 1:**
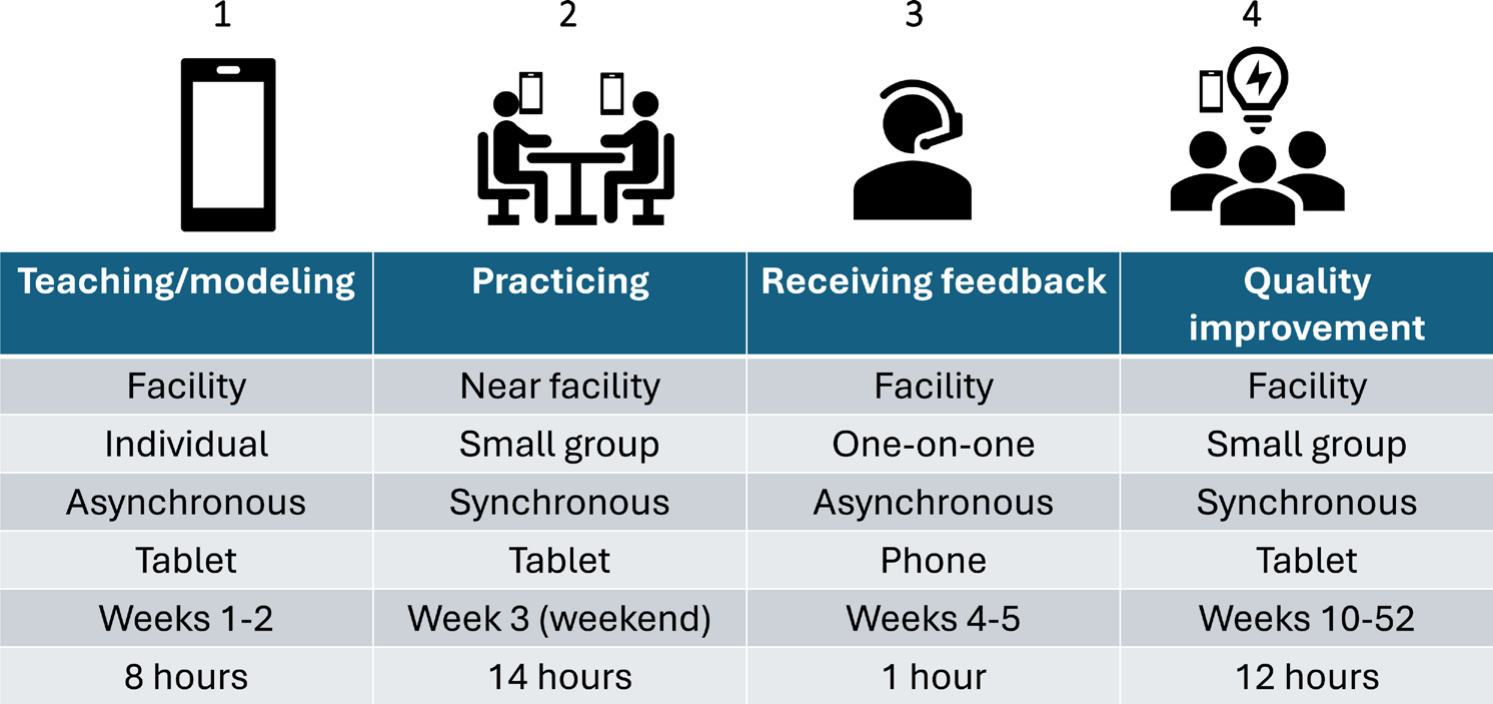
Enhanced implementation package.

In addition to these standard implementation activities, the 11 clusters assigned to the enhanced arm received a digitally-based intervention (figure 1). This package was guided by formative qualitative research with health workers^25^ and the consolidated framework on implementation research^20 26^ and has been described in detail.^21^ Briefly, it consisted of:

1. Teaching and role-modelling: this consisted of five individual asynchronous tablet-guided sessions that health workers completed during work hours. They took 8 hours and were completed over 3 weeks. They focused on index case testing principles, basic counselling skills and the guidelines for counselling index and contact clients. A narrator described protocols, and actors modelled counsellor and client roles. At each facility, a Tingathe site supervisor ensured session completion without clinical disruption.
2. Practising: these activities were designed to assimilate knowledge and skills learnt in the teaching and role-modelling sessions. Activities consisted of eight small-group synchronous sessions that were tablet-guided and conducted over a full weekend. Health workers took turns practising skills based on a set of prespecified scenarios.
3. Receiving feedback: individualised feedback was provided through a one-on-one phone call with a study staff member that lasted 45 min. The study staff member pretended to be a client based and the health worker was asked to counsel them. They were given feedback.
4. Improving quality: a group of health workers periodically examined their facility’s index case testing indicators, identifying challenges and working towards solutions. A tablet was used to guide health workers through a structured process. This occurred six times over 1 year; each lasted 2 hours.

### Data collection

Cluster-level characteristics were collected through a baseline facility survey. Training data were ascertained through digital records, which were collected through SurveyCTO. Facility-level data were abstracted from MOH index and contact registers. Effectiveness outcomes were measured as a count per cluster per calendar-quarter (3-month period). Cluster-level rather than individual-level data were used because the clusters were the primary unit of randomisation and consenting individual-level data were not available. Adverse events were reported through an unanticipated adverse event form.

### Outcome measures

Adoption of the enhanced implementation strategy was measured as the proportion of health workers in the enhanced arm who completed each session. We report the median and range for each of the four components.

To examine effectiveness, we examined the impact of the enhanced implementation strategy on five coprimary clinical indicators at the cluster level: (1) rate of index clients who participated, (2) rate of contact clients elicited, (3) rate of contact clients with recorded HIV testing, (4) rate of contact clients newly diagnosed with HIV and (5) rate of HIV self-test kits distributed. For each of these outcomes, facility-level data were abstracted from programmatic index testing registers on a quarterly basis. For each rate, the numerator was measured as a count per cluster per calendar-quarter. The denominator was the number of potential index clients per cluster per calendar-quarter. Potential index clients were estimated as the number of people in the ART and HTS settings expected to receive index case testing services in a calendar-quarter. In the HTS setting, this consisted of people with HIV-positive diagnoses. In the ART setting, this consisted of people presenting for HIV treatment. We examined indicators by setting (HTS and ART) both separately and overall.

All adverse events were reported and captured on study forms with the date and time, location, nature, severity and relatedness to the study. Events were reported to regulatory agencies and participants were referred to appropriate services.

### Analyses

As described, we compared the enhanced versus standard arms for rates of five clinical outcomes using cluster-level data from four calendar-quarters (a 1-year period). For each of the five programmatic outcomes, we estimated a rate ratio (RR) and corresponding 95% CI using a negative binomial mixed-effects model accounting for repeated measures within cluster. Each negative binomial model was fit using a log link and included a parameter for randomisation arm, the natural-log transformed number of potential index clients as a statistical offset term, randomisation strata and a random intercept for cluster. Analyses were conducted separately within the HTS and ART settings (subanalyses) as well as overall (main analysis). Analyses were conducted in SAS V.9.4 (Cary, North Carolina) or R (V.4.4.2). A type 1 error rate of 0.05 was used throughout, without adjustment for multiple outcomes, in accordance with our study protocol.^21^

Statistical power and sample size calculations were conducted using simulation studies based on 2019–2020 background data from the 33 clusters. We demonstrated 80%–85% power to detect a difference between the enhanced and standard arms for each of the five coprimary clinical outcomes.^21^

## Results

**Figure 2:**
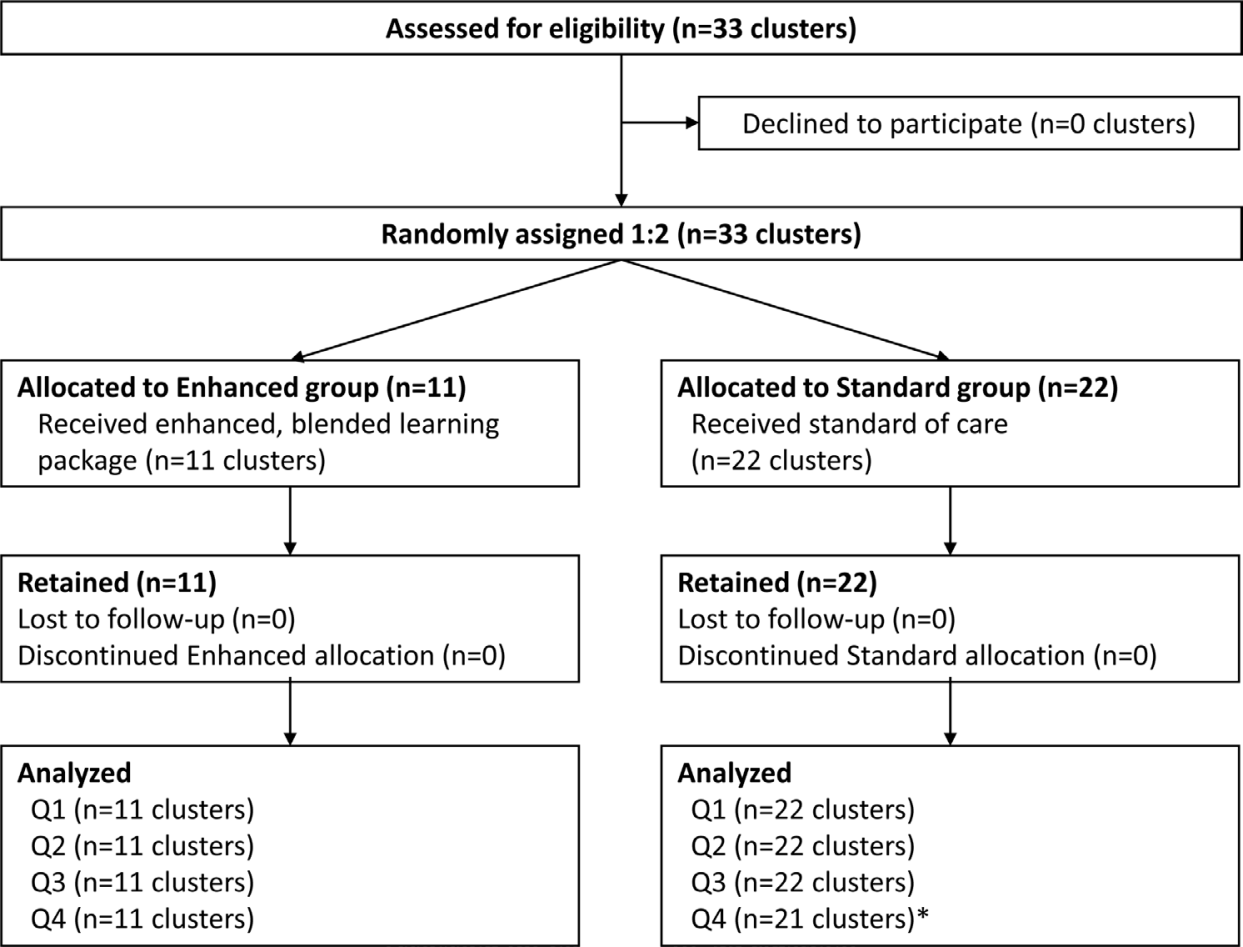
CONSORT diagram. *In Q4 of the study period, two facilities in the standard group merged their HIV testing services and thus were pooled into one cluster for analysis. CONSORT, Consolidated Standards of Reporting Trials. Q1-4, calendar-quarters one to four in the study year.

Thirty-three clusters were eligible, included and randomly assigned: 11 to the enhanced arm, 22 to the standard arm (figure 2). All were retained and analysed in their allocated arm over the course of the full year (September 2022–October 2023). One standard cluster was shut down for maintenance in the final quarter and merged with a nearby standard facility. These two facilities were analysed as one observation, rather than two, during this final quarter. Thus, there were 131 cluster-quarter observations in the final dataset (n=(33 clusters×4 quarters)−1 merged).

Twenty clusters were in Machinga and 13 were in Balaka (table 1). Twenty-eight were health centres, three were dispensaries and two were hospitals. Twenty-six were rural and seven were peri-urban or urban. Twenty-three were operated by the Government of Malawi under the Ministry of Health (MOH); 10 were operated by the Christian Health Association of Malawi. All implemented the MOH HIV programme. HTS and community-based tracing activities were offered on all weekdays. Adult ART services were offered a median of 4 days each week and paediatric ART services 1 day per week. Baseline characteristics were similar between arms.

**Table 1:** Facility characteristics at baseline (n=33 clusters)

Characteristic	Enhanced (n=11)	Standard (n=22)
District, n (%)		
Balaka	4 (36%)	9 (41%)
Machinga	7 (64%)	13 (59%)
Facility type, n (%)		
Dispensary	1 (9%)	2 (9%)
Health centre	9 (82%)	19 (86%)
District hospital	1 (9%)	1 (5%)
Facility location, n (%)		
Peri-urban/urban	2 (18%)	5 (23%)
Rural	9 (82%)	17 (77%)
Facility ownership, n (%)		
Christian Health Association of Malawi (CHAM)	4 (36%)	6 (27%)
Ministry of Health	7 (64%)	16 (73%)
Facility estimated catchment area, n (%)		
<20 000	3 (27%)	8 (36%)
20 000-49 999	7 (64%)	10 (45%)
>50 000	1 (9%)	4 (18%)
Number of healthcare workers, median (IQR)	7 (6, 10)	7 (5, 9)
Number of days per week offering HIV testing services, median (IQR)	5 (5, 6)	5 (5, 6)
Number of days per week offering adult ART services, median (IQR)	4 (1, 5)	4 (2, 5)
Number of days per week offering paediatric ART services, median (IQR)	1 (0, 2)	1 (1, 3)
Number of days per month offering community-based physical tracing, median (IQR)	20 (12, 20)	20 (15, 20)

IQR (25th–75th percentile).

ART, antiretroviral therapy.

All health workers recruited were screened, found eligible and provided informed consent. A median of seven health workers enrolled at each facility (IQR: 6–9). There were 125 health workers in the enhanced arm and 181 in the standard arm.

Adoption of the implementation package was high. In the enhanced arm, 100% of health workers completed each teaching and role modelling session, 98% completed each practice session, 100% completed the feedback session and a median of 89% completed each quality improvement session. In the standard arm, none of the health workers completed any sessions.

### ART setting outcomes

In the ART setting, the median number of potential index clients was 265 per cluster in each calendar-quarter (IQR: 206–400) (table 2). The median rates of index client participation were 0.52 in the enhanced arm and 0.43 in the standard arm (RR): 1.27, 95% CI 0.93 to 1.75, p=0.1) (table 2). The rate of contact client elicitation was higher in the enhanced arm compared with the standard arm (0.1 vs 0.06; RR: 1.68 (CI 1.16 to 2.44), p=0.007). Comparing the enhanced to the standard arm, the rate of contact clients tested was 1.93 (95% CI 1.27 to 2.91, p=0.002) times higher, the rate of contact clients diagnosed with HIV was 2.55 (CI 1.43 to 4.52, p=0.002) times higher, and the rate of HIV self-test kits distributed to index clients was 2.97 (CI 1.12 to 7.89, p=0.03) times higher (table 2).

**Table 2:** Clinical outcomes (n=131 calendar-quarters)*

	Median counts (IQR)		Median rates (IQR)†		Rate ratio (95% CI)	P value
	Enhanced	Standard	Enhanced	Standard		
ART setting						
Potential index clients	237 (187, 398)	269 (219, 416)				
Index clients who participated in ICT	125 (87, 174)	121 (54, 186)	0.52 (0.29, 0.86)	0.43 (0.21, 0.67)	1.27 (0.93 to 1.75)	0.1
Contact clients elicited	35 (19, 51)	16 (8, 29)	0.10 (0.06, 0.18)	0.06 (0.03, 0.10)	1.68 (1.16 to 2.44)	0.007
Contact clients tested	17 (11, 39)	10 (5, 19)	0.05 (0.03, 0.14)	0.04 (0.01, 0.07)	1.93 (1.27 to 2.91)	0.002
Contact clients diagnosed HIV+	1 (0, 2)	0 (0, 1)	0.0002 (0, 0.0049)	0 (0, 0.0023)	2.55 (1.43 to 4.52)	0.002
HIV self-test kit to index client	3 (1,5)	0 (0, 4)	0.009 (0.003, 0.018)	0 (0, 0.011)	2.97 (1.12 to 7.89)	0.03
HTS setting						
Potential index clients	18 (13, 28)	19 (13, 28)				
Index clients who participated in ICT	17 (14, 26)	19 (13, 28)	1.00 (0.93, 1.05)	0.95 (0.88, 1.00)	1.04 (0.95 to 1.14)	0.4
Contact clients elicited	23 (16, 36)	20 (14, 33)	1.24 (1.00, 1.65)	1.06 (0.83, 1.40)	1.20 (0.97 to 1.48)	0.09
Contact clients tested	14 (10, 24)	14 (8, 20)	0.75 (0.41, 1.35)	0.64 (0.33, 1.00)	1.18 (0.88 to 1.58)	0.3
Contact clients diagnosed HIV+	1 (0, 2)	1 (0, 2)	0.04 (0, 0.09)	0.05 (0, 0.10)	1.00 (0.67 to 1.49)	>0.9
HIV self-test kit to index client	2 (1, 4)	1 (0, 2)	0.09 (0.03, 0.19)	0.05 (0.00, 0.11)	1.93 (1.09 to 3.40)	0.02
Both settings combined						
Potential index clients	249 (200, 421)	286 (229, 443)				
Index clients who participated in ICT	140 (114, 189)	136 (79, 211)	0.56 (0.33, 0.87)	0.46 (0.30, 0.68)	1.22 (0.93 to 1.60)	0.1
Contact clients elicited	57 (42, 84)	42 (25, 62)	0.19 (0.13, 0.27)	0.13 (0.09, 0.19)	1.37 (1.10 to 1.71)	0.006
Contact clients tested	38 (25, 56)	27 (17, 35)	0.11 (0.06, 0.20)	0.08 (0.05, 0.11)	1.45 (1.10 to 1.92)	0.01
Contact clients diagnosed HIV+	2 (1, 4)	2 (1, 3)	0.005 (0.002, 0.008)	0.004 (0.003, 0.008)	1.28 (0.94 to 1.76)	0.1
HIV self-test kit to index client	5 (3, 10)	1 (0, 7)	0.016 (0.008, 0.028)	0.006 (0.000, 0.016)	2.29 (1.19 to 4.40)	0.01

IQR (25th–75th percentile).

* There were four calendar-quarters per cluster (n=33) from October 2022 to September 2023. The enhanced arm had 11 clusters and 44 observations. The standard arm had 22 clusters and 87 observations.

† Quarterly rates were calculated as outcome counts per the number of potential index clients.

ART, antiretroviral therapy; HTS, HIV testing services; ICT, index case testing programme.

### HTS setting outcomes

In the HTS setting, the median number of potential index clients was 19 per cluster in each calendar-quarter (IQR: 13–28). The rate of index client participation did not differ in the enhanced and standard arms (median rates: 1.00 vs 0.95; RR: 1.04 (CI 0.95 to 1.14), p=0.4). The estimated contact client elicitation rates were 1.24 (enhanced arm) and 1.06 (standard arm) (RR: 1.20, CI 0.97 to 1.48, p=0.09). There was not a discernible effect on contact client testing (RR: 1.18, 95% CI 0.88 to 1.58, p=0.3) or contact client HIV diagnosis (1.00, 95% CI 0.67 to 1.49, p>0.9). The median number of HIV self-test kits provided to index clients for secondary distribution was higher in the enhanced arm than the standard arm (RR: 1.93, CI 1.09 to 3.40, p=0.02).

### Primary outcomes

**Figure 3:**
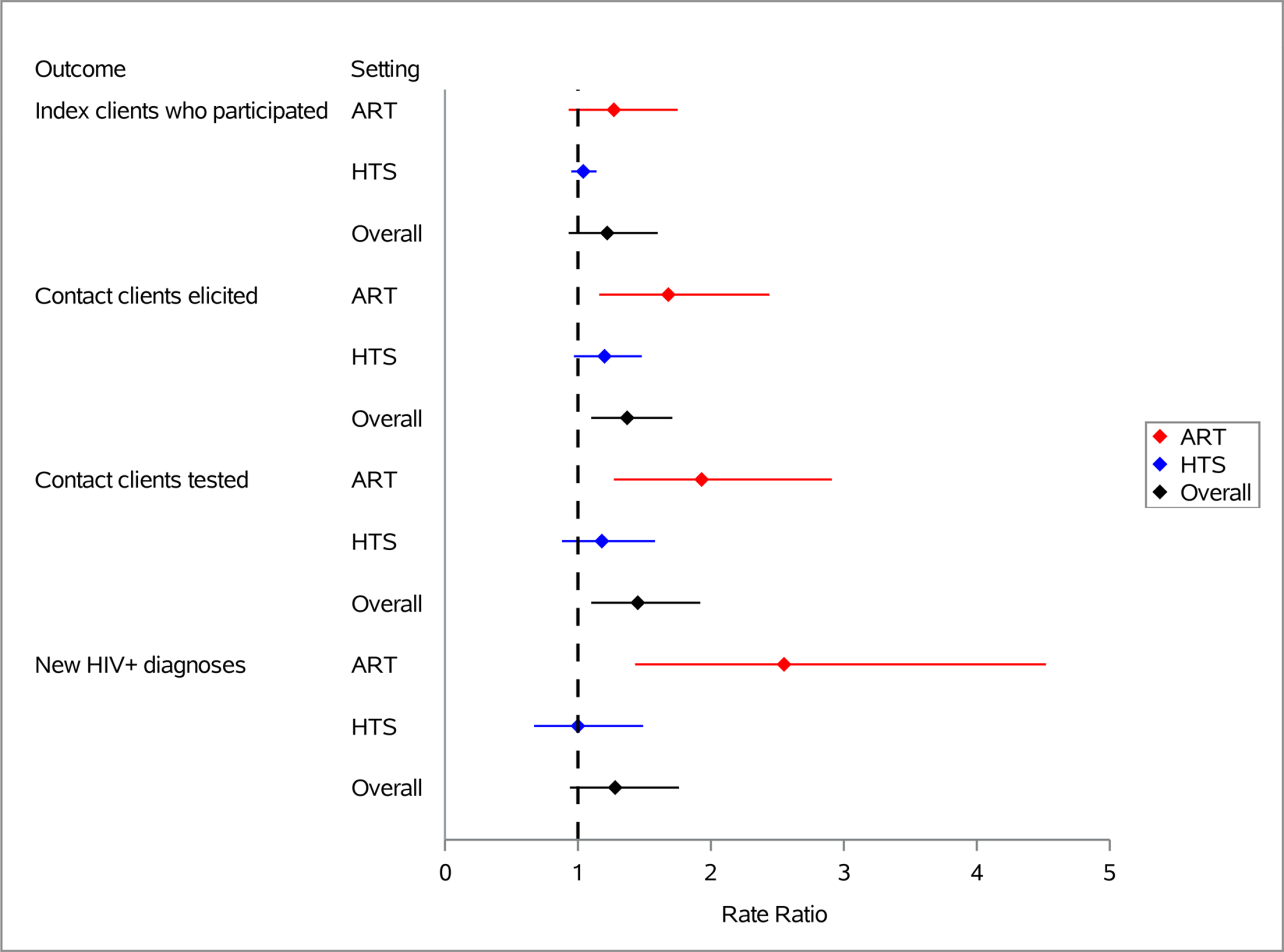
Impact of enhanced implementation strategy on HIV index case testing clinical outcomes. ART, antiretroviral therapy; HTS, HIV testing services.

Combining the ART and HTS settings together, we observed higher index client participation in the enhanced arm (RR: 1.22, CI 0.93 to 1.60, p=0.1), but this result was not statistically significant (figure 3). There were significantly more contact clients elicited (RR: 1.37, CI 1.10 to 1.71, p=0.006), contact clients tested (RR: 1.45, CI 1.10 to 1.92, p=0.01), and self-test kits distributed (RR: 2.29, CI 1.19 to 4.40, p=0.01) in the enhanced arm compared with the standard arm (table 2).

For contact clients diagnosed with HIV, the estimated effect was encouraging for the enhanced strategy, though not statistically significant (RR: 1.28, CI 0.94 to 1.76, p=0.1).

### Adverse events

One health worker in the enhanced arm died due to a health complication unrelated to study participation. There were no study-related adverse events.

## Discussion

Across two Malawian districts, the PRACTICE study examined the impact of an enhanced implementation strategy delivered to health workers on a range of HIV index case testing clinical indicators. In the overall programme, we observed positive effects on contact client elicitation, contact client HIV testing and provision of self-test kits for secondary distribution. Additionally, higher rates of index client participation and new HIV diagnoses were encouraging, though not statistically conclusive. Health workers in the enhanced arm adopted nearly the entire package. There were no study-related adverse events.

The enhanced training package had a greater impact in the ART setting than in the HTS setting. In the HTS setting, in both arms, nearly all potential index clients received index case testing services, leaving little room for improvement. In contrast, in the ART setting, in the standard arm, a median of 43% of index clients received index case testing services; this median level improved to 52% in the enhanced arm. Additionally, the enhanced package had a substantial impact on contact client elicitation in the ART setting and a modest impact in the HTS setting. This was likely due to health workers in the enhanced arm being more thorough and skilled when counselling index clients.^27^ Higher index client participation and greater contact client elicitation led to more contact clients overall, and thus more being tested for HIV and ultimately a few more diagnoses.

The absolute number of new HIV diagnoses was small—a median of two diagnoses per cluster per calendar-quarter in both study arms. This low value was an artefact of a mature HIV epidemic. During the study period, nationally 9% of adults were living with HIV and only 12% of them did not know their HIV status.^24^ Thus, only 1% of adults in the general population were living with HIV and unaware of their HIV status.^24^ In contrast, approximately 6% of those tested in our study were living with HIV and unaware of their status, reinforcing the high HIV diagnostic yield from index case testing. Although these yields were lower than those in earlier stages of the HIV epidemic,^28^ they remained an efficient way of finding people in need of HIV diagnosis.

Other than our pilot assessment,^20^ this is the first study to rigorously examine a health worker-level implementation strategy in the index case testing setting. Most research has examined implementation strategies at the client level, such as degree of clinical support or testing modality. Thus, our work offers a novel contribution to both the HIV testing and implementation science fields.

Our implementation strategy was developed in response to several observations in Malawi’s HIV index case testing programme. We observed that index case testing outcomes in routine settings were considerably worse than those in foundational trials and demonstration projects.^28^ In our formative work, health workers expressed a need to observe, practise and get individualised feedback to improve skills.^26^ The digital platform allowed us to roll this out at scale.

Our entire package was taken up by nearly all health workers. Although digital and blended learning have become routine in many high-income settings, they pose unique feasibility challenges in low-income settings, especially those with low internet connectivity, smartphone penetration and digital literacy. We developed our implementation strategy with these constraints in mind on programme-owned tablets, with an *offline* option, and simple navigation. Without these design considerations, adoption would likely have been much lower.

Digital learning has many appealing features. First, high-quality content can be delivered consistently. Second, disruptions to clinical activities are minimised, as asynchronous activities are staggered and synchronous activities occurred on weekends. Had this 4-day training taken place in a more traditional way (facilitator-delivered at a hotel), each healthcare worker would have missed a week of clinical activities. Third, we were able to deliver 4 days of training at a lower cost. A comparable amount of training offered synchronously at an off-site location would have cost at least $500 per healthcare worker in allowance, food and lodging costs. Finally, we were able to measure and monitor the ‘dose’,” that each health worker received, using completion records.

These cost and feasibility features are especially appealing in the new global funding landscape.^29^ As development assistance for health diminishes, opportunities to provide in-service training at lower cost are becoming ever more critical to sustainable programming. A next step is to develop a generative artificial intelligence tool to provide this feedback. Such opportunities were not possible when developing our implementation package in 2021, but have become an intriguing possibility in the ensuing years.^30^,^32^

The decision to conduct a pragmatic trial has strengths and limitations. Our findings have high external validity, capturing the entire index case testing programme in two districts and a range of facility types. We used routinely collected clinical data, representing approximately 50 000 people living with HIV. This decision not only minimised selection bias but also had limitations. We did not have index-level and contact client-level information, such as individual HIV testing history, time on treatment or past experiences with index case testing. Similarly, we could not link index clients to their contacts, making linked analyses impossible.

A second limitation stems from having five primary outcomes without multiplicity adjustment. Statistical opinions differ surrounding the handling of multiplicity in pragmatic trials^33^ and multiplicity adjustment practices vary.^34^ We decided not to adjust for multiplicity because this practice can obscure meaningful findings by artificially inflating the significance threshold.

A final limitation is the inability to isolate which components of the implementation strategy drove effectiveness. Nearly all participants completed most activities, thus we do not know whether certain components drove impact or whether they all worked synergistically.

## Conclusions

Often, health worker-level implementation strategies are developed and offered but not rigorously evaluated. Our cluster randomised trial had a rigorous design and long-term follow-up. Furthermore, we observed excellent implementation and improvements in three meaningful clinical outcomes. Together, these design features allowed us to make an important and novel contribution, demonstrating that even in a setting with poor internet connectivity and relatively low digital literacy, an intensive digitally-guided training package could improve HIV testing outcomes. These findings have implications for other training programmes, where digital learning offers an enticing opportunity for building lay health worker capacity, a necessary step towards the sustainable development goals.

## Supplementary material

10.1136/bmjgh-2025-022563 online supplemental file 1

## Data Availability

Data are available in a public, open access repository.
