# Supplementary material for: Effect of a digital training package on clinical outcomes in Malawi’s index case testing programme: a cluster randomised controlled trial
Source: BMJ Glob Health. 2026 Mar 13;11(3):e022563. doi: 10.1136/bmjgh-2025-022563 (PMC12993308; doi:10.1136/bmjgh-2025-022563)
Supplement: online supplemental file 1 [file bmjgh-11-3-s001.docx]

### BMJ Global Health Author Reflexivity Statement

Adapted from Morton, B., Vercueil, A., Masekela, R., Heinz, E., Reimer, L., Saleh, S., Kalinga, C., Seekles, M., Biccard, B., Chakaya, J., Abimbola, S., Obasi, A. and Oriyo, N. (2022), Consensus statement on measures to promote equitable authorship in the publication of research from international partnerships. Anaesthesia, 77: 264-276. <https://doi.org/10.1111/anae.15597>

| **Study conceptualisation** | |
| --- | --- |
| 1. How does this study address local research and policy priorities? | This study directly supports Malawi’s Ministry of Health HIV testing and treatment priorities by evaluating scalable approaches to assisted contact tracing (ACT), a key element of the national HIV strategy and WHO “95-95-95” targets, and also informed on the feasibility of digital training in LMIC. |
| 1. How were local researchers involved in study design? | Baylor Foundation Malawi leadership and Malawi-based investigators contributed to protocol design, site selection, and implementation strategies, ensuring feasibility and alignment with national ACT rollout plans |
| **Research management** | |
| 1. How has funding been used to support the local research team(s)? | NIH R01 funding was used to employ Malawi-based Baylor Foundation Malawi staff, provide field tablets and supplies, cover local investigator salaries, and support data management infrastructure at site. |
| **Data acquisition and analysis** | |
| 1. How are research staff who conducted data collection acknowledged? | All Baylor Foundation Malawi field staff, supervisors, and data clerks involved in ACT implementation are acknowledged in study reports and manuscripts, and key contributors are included as co-authors. |
| 1. How have members of the research partnership been provided with access to study data? | Both UNC and Baylor Foundation Malawi have full access to the study database through secure shared servers, with de-identified data accessible to Malawi investigators for analysis and local reporting. |
| 1. How were data used to develop analytical skills within the partnership? | Joint analytic workshops and mentoring sessions between UNC and Baylor Foundation Malawi staff were conducted to strengthen local capacity in implementation-science methods, costing, and qualitative analysis. |
| **Data interpretation** | |
| 1. How have research partners collaborated in interpreting study data? | Results interpretation is led jointly by UNC and Baylor Foundation Malawi investigators through regular virtual meetings where KUHEs and MoH representatives were involved, with Malawi team members providing contextual insights critical for accurate interpretation. |
| **Drafting and revising for intellectual content** | |
| 1. How were research partners supported to develop writing skills? | Manuscript development follows a mentored authorship model pairing junior Baylor Foundation Malawi staff and KUHEs with UNC investigators for structured writing support and iterative feedback. |
| 1. How will research products be shared to address local needs? | Findings will be disseminated through Malawi Ministry of Health dissemination meetings, Baylor Foundation Malawi’s annual review sessions, and open-access publications targeting implementers and policymakers. Local dissemination meeting including all key stakeholders in Malawi, are planned to disseminate at the facilities, MOH technical working groups, international and local conferences. |
| **Authorship** | |
| 1. How is the leadership, contribution and ownership of this work by LMIC researchers recognised within the authorship? | Malawi-based investigators serve as site PI and senior co-authors, reflecting their leadership in study design, implementation, and interpretation. |
| 1. How have early career researchers across the partnership been included within the authorship team? | Early-career staff and trainees from both UNC and Baylor Foundation Malawi are included as co-authors for their contributions to data collection, analysis, and writing. |
| 1. How has gender balance been addressed within the authorship? | The authorship team includes balanced representation of women and men across senior and junior roles, reflecting the gender composition of the study leadership team. |
| **Training** | |
| 1. How has the project contributed to training of LMIC researchers? | The project provides on-the-job training in implementation research, data analysis, manuscript development, presentation and dissemination skills for Baylor Foundation Malawi and Ministry of Health staff, enhancing national research capacity. Site study investigators were also supported to attend regional and international conferences for additional training. |
| **Infrastructure** | |
| 1. How has the project contributed to improvements in local infrastructure? | Funds supported procurement of tablets, secure data storage servers, desks and charis at the office, laptops for workstation, vehicles for travel, and internet connectivity improvements in study districts, which remain available for subsequent Ministry and Baylor Foundation Malawi projects. |
| **Governance** | |
| 1. What safeguarding procedures were used to protect local study participants and researchers? | The study received ethical approvals from UNC-Chapel Hill and Malawi’s NHSRC, used coded identifiers for all participants, and conducted staff training on confidentiality, data security, and psychosocial safety during partner tracing. |
